# The journey to reporting child protection violations in sport: Stakeholder perspectives

**DOI:** 10.3389/fpsyg.2022.907247

**Published:** 2023-01-04

**Authors:** Yetsa A. Tuakli-Wosornu, Sandi L. Kirby, Anne Tivas, Daniel Rhind

**Affiliations:** ^1^Department of Social and Behavioral Sciences, Yale School of Public Health, New Haven, CT, United States; ^2^Department of Physical Medicine and Rehabilitation, University of Pittsburgh School of Medicine, Pittsburgh, PA, United States; ^3^Department of Sociology, University of Winnipeg, Winnipeg, MB, Canada; ^4^Safe Sport International, London, United Kingdom; ^5^School of Sport, Exercise, and Health Sciences, Loughborough University, Loughborough, United Kingdom

**Keywords:** safe sport, human rights, athletes, grievance reporting, emotional abuse, sexual abuse, physical abuse, neglect

## Abstract

Sport is a context within which human and children’s rights should be respected, promoted, and protected. Yet, research and high-profile cases demonstrate that this is not always the case. To understand the existence (or not) of reporting mechanisms for child protection violations in sport, as well as how existing reporting and response systems operate, the authors, with the support of the Centre for Sport and Human Rights, conducted research on current abuse disclosure and reporting pathways in sport. The purpose was two-fold: to describe global child protection systems and reporting mechanisms, and to identify major areas of stakeholder concern, in terms of effective case resolution, healing, and children’s experiences along reporting pathways in sport. Two sources of evidence were tapped. First, a rapid evidence assessment consisting of a literature review and an exploratory survey with 112 global stakeholders was conducted. Second, focus group interviews informed by the evidence assessment were held with nine athletes with lived experiences of abuse in youth sport and 13 global human and children’s rights experts primarily working outside of sport. Through this emergent research, a ‘pathway’ or ‘journey’ to incident reporting in sport was developed, summarized as 5 ‘Rs’: **R**eadiness, **R**ecognition, disclosure and **R**eporting, **R**esponse, and **R**emedy, which are similar but not identical to existing trauma frameworks. Each stage of the reporting journey appears to be influenced by a range of contextual, organizational, relational, and individual factors. All told, the disclosure of child protection violations in sport is a complex and dynamic process where myriad factors interact to influence outcomes, including healing. Key recommendations include: (a) establishing a global Safety Net Environment in sport practice with varying applications from region to region, (b) building bridges with specific partner organizations to enhance child protection and safeguarding work in sport and (c) bringing safeguarding to unregulated sporting environments.

## Introduction

Often positioned as an antidote to inter- and intrapersonal distress, sport is increasingly recognized as a context that can contain interpersonal violence.^1^ This and other human and children’s rights violations have been evidenced by powerful testimonies, growing research evidence, case analyses and international inquiries emerging from diverse sport contexts (Lang and Hartill, 2014; Kaufman et al., 2022). Concern regarding such violations has been exacerbated by the global pandemic which has had wide-ranging implications for the protection and promotion of human rights in, around, and through sport (Rozga et al., 2020). As a result, it is critical that a comprehensive understanding of how, when, where, and why interpersonal violence against children in sport can be reported. To facilitate a joined-up approach to understanding violence, rights, and sport, and in the context of “firmly establishing the relationship between child rights and playing sport, and sport and the protection and fulfilment of rights” (Rozga et al., 2020), this study addresses barriers to and facilitators of reporting child protection violations in sport.

Of note, sexual violence is the type most talked about in historical literature from the sports sector. Therefore, our discussion gravitates slightly toward sexual violence but is not intended to exclude other forms. Additionally in this paper, we place emphasis on children and young people. That is because the foundational work in this space focuses exclusively on this group (David, 1998, 2004). We do however acknowledge that in sport, and with regards to human rights, strict distinctions between children and adults is somewhat artificial, since human rights apply to all, and “age boundaries are irrelevant in a field of activity like sport where elite performers might be in their low teens and beginners as old as 80 (Brackenridge in Lang and Hartill, 2014).”

### Contextualizing child abuse in sport

Much global progress has been made in the area of violence prevention in youth sport (David, 2004). Outside the sports sector, literature concerning violence prevention and other human rights protections for children has matured, particularly considering key developments such as the United Nations’ Convention on the Rights of the Child (UNCRC) (1989); the 2006 study on Global Violence Against Children (Pinheiro, 2006); and the United Nations Educational, Scientific and Cultural Organization (UNESCO) mandate of building peace through international cooperation, which covers child development and the creation of safe and respectful environments in which to live and play.

What is clear from the mainstream child protection literature is that children at various stages of emotional, physical, and cognitive development, find it extremely difficult to recognize, talk about, and report their experiences of interpersonal violence. Safe Sport International (SSI), with which all authors affiliate, works from the premise that it is adults’ responsibility to create safe sports environments, identify indicators that a child may be experiencing abuse and report these. Adults who work in sport also need to raise children’s awareness of their rights in developmentally appropriate ways and provide them with information about who they can turn to if they have worries (Brackenridge et al., 2010; Lang and Hartill, 2014). In this way, child protection is sports’ responsibility, not childrens’.

Despite the benefits of co-production in developing effective sport practices (Smith et al., 2022), SSI’s founding members have observed over the past 20 years that children are rarely involved as key stakeholders in sport organizations’ safeguarding work. This is slowly changing (Lang, 2022), but from our considerable experience, sport appears slow to hear children’s voices and may lack confidence in doing so.

David’s (1998) *Children’s Rights and Sport* report, and his 2004 *Human Rights in Youth Sport* manuscript were initial bridging points between human rights and youth sport. These worlds came even closer together when in 2006, the United Nations’ Children’s Fund (UNICEF) Innocenti Centre invited sport researchers, policy makers and practitioners led by Professors Celia Brackenridge and Kari Fasting for a consultation on violence against children in sport. This led to the subsequent UNICEF *Review of Violence against Children in Sport* (Brackenridge et al., 2010). In 2014, the *International Safeguards for Children in Sport* initiative was launched in Johannesburg South Africa, providing eight general guiding principles for organizations who work with children (Rhind and Owusu-Sekyere, 2017; Rhind and Owusu-Sekyere, 2020). A number of these recommendations emphasized the contextual, organizational, and cultural drivers of abuse in youth sport.

The International Olympic Committee (IOC) invited a similar group to the earlier UNICEF gathering, to deliver a *Consensus Statement on Sexual Harassment and Abuse in Sport* in 2006. Second and third versions were produced in 2007 and 2016, the latter citation based on considerably more research, focused on all forms of interpersonal violence, and was explicitly attentive to special populations: children competing at elite levels, Para athletes, and LGTQ athletes (Mountjoy et al., 2016). All told, work from 1998 onwards has underlined that successful cooperation across mandates is clearly the path forward in raising awareness about abuse prevention in youth sport.

Sport has not yet developed a coherent global child protection, reporting, and response framework or system, which could potentially help promote, coordinate, and monitor sport safeguarding concerns in a similar way that the World Anti-Doping Agency addresses athlete doping. There is no evidence to confirm that such an approach would be effective or ideal (Tuakli-Wosornu et al., 2021a), but it could help raise global safeguarding awareness. National surveys related to grievance reporting mechanisms have been conducted among athletes in Canada (Kirby and Greaves, 1996; Kirby et al., 2000; Parent et al., 2016; Kerr et al., 2019; Parent and Vaillancourt-Morel, 2020); Germany (Stadler et al., 2012; Ohlert et al., 2018); Norway (Fasting et al., 2004); the United Kingdom (Alexander et al., 2011), Zambia (Fasting et al., 2015) and Netherlands and Belgium (Vertommen et al., 2016). The global South (Africa, South America, Asia) is not well-represented in such analyses. No comprehensive data yet exists on the types of abuse reporting mechanisms that are in place nor how effective they are in youth sport.

### Reporting issues for adults who observed abuse or were abused as children

Research by Guiora (2020) has highlighted the institutional complicity and betrayal young athletes experience when bystanders/enablers do not report abuse even when the adults in question (athletes, coaches, parents, and other stakeholders) are direct witnesses to it or informed directly about it. For some athletes, even more traumatizing than the abuse itself was the fact that adults or authority figures clearly knew about their abuse as children and either did nothing or actively sent young athletes toward abusers. The 2021 Project CARE report demonstrated how devasting adult inaction in response to abuse disclosures from children can be: “I told my coach about it (sexual harassment), nothing was done. You know, the Federation knew about this guy doing things, and nothing was done. So the story of my career was, nothing was done to correct for the injustices like this (Rhind et al., 2021).” Vertommen and her team in 2019 showed that even indirect *signs* of child abuse in sport are often not acted upon. Most of the extant data on adults in sport deciding not to report child abuse emerge from institutional or academic abuse inquiries such as national independent inquiries into sexual abuse in sport (Kerr et al., 2019; Rhind et al., 2021; Sheldon, 2021), and emphasize the toxic culture of silence that is cultivated in youth sport when direct abuse disclosures and indirect signs of abuse are ignored.

Additional insights and theories as to why adults (athletes, coaches, teammates, and other stakeholders) may not report concerns and/or may perceive barriers to reporting concerns about abuse in youth sport settings have been put forward. The perception that children are vulnerable and incompetent (Lang, 2022), as well as failure to appropriately identity harmful behavior, low self-efficacy for reporting, steep coercive power imbalances, and strict social hierarchies have been identified as factors that promote inaction/non-reporting (Vertommen et al., 2019; Verhelle et al., 2022). In a recent study out of Zambia (Solstadt, 2019), lack of social support, wanting to fit in and poverty-related issues were additionally cited as barriers to action/reporting.

Data on adult athletes who were abused as children in sport are increasingly available. The largest study to examine interpersonal violence in sport within six European countries found the majority of adults who had experienced interpersonal violence as children in sport never reported their most serious experience, rarely disclosed to someone inside sport, and if they did disclose, most commonly disclosed to family members or relatives, friends, peers or personnel in education and health (Hartill et al., 2021). Data concerning *why* this cohort does not disclose harms are somewhat inconclusive, in part due to heterogeneous methodology and definitions of harm. Still, research shows that key disclosure-related variables include when the abuse was experienced, the nature of the abuse (physical, psychological, sexual, etc.), the victim’s gender (Briere and Conte, 1993; Hartill et al., 2021), and children’s awareness of their agency (Rhind et al., 2021). Project CARE found that 69% of adult athletes surveyed were not always aware they had any ‘rights’ when they were children in sports, and Tuakli-Wosornu found in 2022 that athletes’ confidence in their rights-related agency and autonomy is both low in sport settings, and strongly influenced by gender (Tuakli-Wosornu et al., 2022b).

In non-sport contexts, early research showed that long-term abuse, abuse at an early age, and particularly violent abuse were not only more difficult to report, but also might not be recalled until adulthood (Briere and Conte, 1993). Adults, including those who have experienced abuse as children, can feel guilt, shame, and even culpability for accepting gifts in exchange for agreeing to sexual relations. These data mirror findings in sport: Leahy et al., 2004 reports that the traumatic impact is higher when emotional manipulation occurs (e.g., coach-athlete abuse). Parent and Vaillancourt-Morel (2020) as well as Toftegaard Nielsen (2001) identify that athletes may perceive some types of abuse as consensual, despite social power imbalances in youth sport. Parent and Vaillancourt-Morel (2020) and Verhelle et al. (2022) also indicate that adults in sport may be uncertain how and where to disclose or how and if the disclosure process would proceed traumatically, or yield any constructive outcome. There is some research to suggest that not only is sexual abuse underestimated in boys/men (Parent and Bannon, 2012), but, that toxic gender norms may influence boys’ abuse disclosure behaviors in sport (Hartill et al., 2021).

The Independent Review into Child Sexual Abuse in Football 1970–2005 (Sheldon, 2021) summarized Operation Hydrant, where some 240 suspects and 692 survivors of child sexual abuse in football, as well as 136 suspects and 201 survivors in other sports were identified. In writing about contemporaneous disclosures, the report indicated that players hinted at their abuse, talked about abuses generally but not their own abuse, and minimized what happened to them and others. Some actually felt they were ‘privileged’ to be targeted/included by the abuser. Shame, not being believed, fear of rocking the boat, worry about ending their dream, and concern about parents retaliating against the abuser kept these young footballers from disclosing. Many survivors were able to hide their abuse for many years, others suffered greatly after their football careers. In football, a sport system of feeder programs continued with little supervision and clubs did not encourage younger players to raise concerns. Even when inappropriate behavior was widely known to be occurring, the young players did not even talk about it among themselves. Silence was an active part of the dynamic of making it into professional football contracts and playing careers (Sheldon, 2021).

### Individual-level risk factors for experiencing abuse in youth sport

Certain factors may be associated with a higher probability of experiencing abuse in sport. Vertommen and her team found in 2016 that people who had a disability, were from an ethnic minority or identified as LGBTQ were significantly more likely to report having experienced abuse in sport. Mountjoy et al. (2016) and Tuakli-Wosornu et al. (2020) identified increased vulnerability to abuse among athletes with disabilities. Parent and Vaillancourt-Morel (2020) found that in Canada, the risk factors for experiencing violence in sport were being a boy, having a greater number of training hours, reporting a non-heterosexual preference, competing at the inter-regional or provincial sport level, and practicing only team sports. Hartill and team in 2021 also found that being a boy increased risk: in the study, the prevalence of interpersonal violence against children inside sport was higher for boys than girls in six European countries. There is also some indication that transitions are times of greater vulnerability for athletes – such as the transition from child to adult, from local/regional teams to international play, and from home location and familial supports to locations far from those (Brackenridge and Kirby, 1997; Kirby et al., 2000).

### Safeguarding response(s) from the international community

Initial *Standards for Safeguarding and Protecting Children in Sport* were developed in 2002 in the United Kingdom. They included requirements for responding to safeguarding concerns, including the provision of reporting mechanisms, and clear information for children about their rights and disclosure processes. In 2014, the *International Safeguards for Children in Sport* were published and brought a strengthened focus on the connection between abuse and rights in sport and sport for development communities’ safeguarding practices, including appropriate responses and remedies (Rhind and Owusu-Sekyere, 2020).

In 2017, the IOC published a Safeguarding Toolkit and requires National Olympic Committees (NOCs) and member International Federations (IFs) to adopt and adapt safeguarding policies and practices. This has occurred unevenly: some NOCs have adopted safeguarding policies and practices, while others have yet to create them. Even in the setting of existing policies and practices, educational developments by many of the IFs (e.g., FIFA, World Rowing) may not be directly aligned with a rights-based understanding of children’s safety. Safeguarding policies do not universally reference children’s rights nor do they place abuse prevention as one part of a holistic view of children’s fundamental human rights (on par with education or healthcare). Clear response and remedy systems are not always articulated in these policies, and to our knowledge, published evaluations of how effective these reporting and response systems have not yet been made available.

The European Union and the Council of Europe have prioritized safeguarding and violence prevention in recent years and have intentionally created programmes which aim to increase the capacity of sport to protect children. The recent *Child Safeguarding in Sport* project aims to increase country sport systems’ capacity to respond to child protection violations through multi-stakeholder development of whole-country roadmaps and the development of child safeguarding officers throughout sport. The role of designated child safeguarding officers is a key development in the sector. While some countries have well-developed processes for handling disclosures and reports of violence against children (not necessarily framed as children’s rights violations) others do not even have a concept of what ‘safeguarding’ might be. There are very few examples of connected sport and statutory agency systems. There is also a paucity of information about appropriate and robust reporting mechanisms for case management. We are unaware of studies that analyse the effectiveness of current reporting mechanisms.

#### Potential bias in international safeguarding responses

Although the UN conventions, specifically UNCRC Section 19: *Protecting Children from Violence*, provide a strong framework for international safeguarding approaches, children’s rights are variously interpreted across cultures – including youth sport cultures (Fay, 2019; Holzscheiter et al., 2019). The UNCRC ‘imposed a universal notion of what it is to be a child; it has prescribed and embedded what the substance and scope of children’s rights should be.” It must be acknowledged that when considering who is a child, how they need protection (in sport and elsewhere) and what is “in their best interests,” the UNCRC takes a distinctly Western view (Fay, 2019; Holzscheiter et al., 2019). Those in the global South may find themselves poorly reflected in Section 19 and others. Local resources, languages, tools and understandings may also limit participation in the critical efforts to decolonize the field of children’s rights and their protections (Faulkner and Nyamutata, 2020; we will rejoin that critique in the discussion section of this paper).

### Conceptual framework and study aim

The 2014 *International Safeguards for Children in Sport* provided the starting point and general conceptual frame for this study. This document was chosen due to its accessibility, easy-to-understand language, and inclusion of pragmatic, actionable recommendations to which those who work outside of sport settings could also relate. In addition, two sections of the document, ‘*Minimising risk to children*’ and ‘*Recruiting, training, and communicating*’ stood out to the authors as an attempt to highlight the importance of contextual and organizational factors, as well as overall culture change, in understanding child protection systems and assessing reporting mechanisms in sport. Contextual drivers of abuse and the impact of organizational culture are central to athlete protection work (Roberts et al., 2020), and we wanted to pay attention to these ideas in this study.

The study aim was to understand the existence (or not) of reporting mechanisms for child protection violations in sport, as well as how existing reporting and response systems operate in order to create a description of global child protection systems and reporting mechanisms, and identify major areas of stakeholder concern, in terms of effective case resolution, healing, and children’s experiences along reporting pathways in sport.

## Materials and methods

A sequential, quantitative-qualitative exploratory study design was used. Survey data provided snapshots of stakeholder experiences and was used to both compare experiential themes with rapid evidence assessment findings and inform the development of the focus group interview guide. Consistent with constructivist approaches (Guba and Lincoln, 2005), the study welcomed discrepancies in the ‘realities’ of different perspectives, experiences and organizations as “any common themes that emerge from great variation are of particular interest and value in capturing core experiences and central shared aspects” (Patton, 1990, p. 172). Ethical approval was granted by the University Ethics Committee of the senior author prior to data collection.

First, a rapid evidence assessment was conducted by the senior author who searched multiple academic databases using terms related to sport, children/youth and abuse, without date limits. This provided an overview of the density and quality of evidence on the issue. Any sources which were relevant to the disclosure of child protection violations in sport were noted and reviewed by the authors, covering all sports and all abuse types.

Based on this, a brief open-ended survey was developed by the senior and middle authors and distributed among 112 global stakeholders by activating and leveraging Centre for Sport and Human Rights’ (CSHR) extensive professional networks. A range of stakeholders were invited to share their views. Five broad survey questions were used, and any information relevant to the disclosure of child protection violations in sport was noted and reviewed by the authors. See Appendix A.

Based on this, focus group consultations were designed in collaboration with CSHR by mapping out the range of parties with relevant expertise on abuse disclosure and reporting systems. Key informants from each stakeholder group were then e-mailed information about the study and invited to participate. The informed consent process followed the senior author’s Institutional Review Board guidelines. Overall, contributions were received from nine athletes with lived experiences of abuse in youth sport and 13 rights experts. A total of 12 distinct countries were represented.

Focus groups were selected as the most appropriate approach to data collection in the context of the global pandemic. These were conducted through online discussions using video calls for practical and logistical reasons. Focus groups enabled interactions and synergies which helped generate data that may not have been possible through other forms of data collection. For each session, the authors prepared an internal ‘agenda,’ made introductions (including key CSHR foci, i.e., prevention of human rights violations, effective remedies, and safeguarding legacies for sport), and shared a prepared diagram for discussion among participants. A diagram was used rather than a more traditional interview guide because the author’s felt that this approach better captured the complexity of the various interacting factors and stakeholders in youth sport. It also enabled participants to focus on aspects of the diagram of greatest interest or relevance to them at the start of the discussion before moving to consider the other areas. This was perceived to facilitate initial discussion flow.

**Figure 1:**
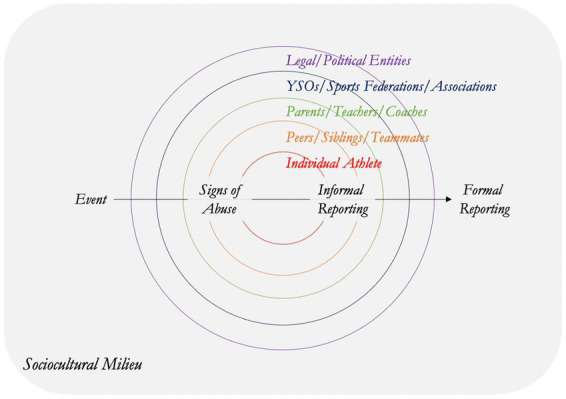
Focus group primer shared with participants in advance of their interview.

Based on an ecological lens, the diagram showed the unique sports environment with layers of entourage groups who can influence participating children, particularly at higher competitive levels. The diagram was developed specifically for the purposes of this research project, and was achieved through serial meetings involving all authors who shared insights, experiences and perspectives (e.g., researcher, elite athlete, medical doctor, social worker and child protection in sport practitioner). The diagram also drew on insights gained through Project CARE which involved interviews with athletes regarding their experiences of disclosing rights violations (Rhind et al., 2021). See Figure 1. The focus groups proceeded as guided discussions on points 1–6 shown at the bottom of Figure 1.

Data were transcribed and analyzed using reflexive thematic analysis (Braun et al., 2016, 2018). Reflexive thematic analysis is a 6-stage process through which themes and patterns within qualitative data are identified, analyzed and reported. Themes were conceptualized from codes as patterns of shared meaning and we inductively analyzed data to identify key stages of the disclosure journey. Results are presented with illustrative participant quotes.

We adopted the trustworthiness criteria outlined by Nowell et al. (2017) to guide our approach. It is argued that this is a pragmatic approach which helps to enhance the acceptability and usefulness of the findings for stakeholders. Firstly, credibility concerns the coherence between the participant’s perspectives and how these are represented by the researchers. This was facilitated through the authors all having prolonged engagement with the context of rights violations in sport. The authors also held regular meetings to reflect on their expectations and experiences such that any biases could be identified and mitigated against. The researchers also engaged with the participants through sharing initial findings and seeking feedback. Secondly, transferability is achieved through providing descriptions of the data and findings to enable others to determine the extent to which the findings can be generalized to their case. Thirdly, dependability was demonstrated through having a traceable process with key stages being recorded. The process was audited by members of the CSHR to check and challenge the procedures used. Finally, confirmability has been demonstrated through justifying our approach at each stage from the background and the project’s rationale through to data collection, analysis and presentation.

Of note, though we frequently used the terms ‘violence’ and ‘abuse’ throughout the project, there are many other terms in use where child protections are enacted. We used different terms when quoting interviewees directly. Additionally, the safeguarding language in sport, while not completely integrated, can differ from language used by those in child protections outside sport (i.e., “non-accidental violence”). Attached project materials such as figures and appendices reveal the variability of terms used among stakeholders with diverse backgrounds, however in the manuscript, we settle into consistent language where possible.

## Results

Data from focus group interviews were analyzed and key themes summarized in relation to two key areas of interest: the reporting journey and the factors which influence this journey. Interviewees included experts on abuse prevention from diverse organizations: End-Violence (*n* = 2), UNICEF (*n* = 7, global), UNICEF Regional Child Protection Advisor (*n* = 1), World Vision International (WVI) (*n* = 1), Child Protection (*n* = 1), End Child Prostitution and Trafficking (ECPAT) (*n* = 1). Exemplar quotes are included to illustrate results.

Generally, stakeholders were largely supportive of the need for this project, and found the exercise valuable as summarized by two sport decision-makers:

“(with the) return to sport” agenda – with COVID – there are higher abuse numbers, increased mental health issues for students and teachers. This is a greater opportunity for integration of safeguarding – holistic, integrative.” *(RH)*

(a) lot of organizations are working to improve children’s access to justice. It is time for the sport system to come in. What new aspects could sport provide to world child protection? We could help them to understand sport environment. Sport should not reinvent the wheel. Sport is not a stand-alone – work with all sectors involved with access to justice. Work with everyone. Form the links. *(FGF)*

Furthermore, it was clear that the consulted experts had deep knowledge of child protection violations, such as sexual abuse, but they had typically not considered the sports context. It is noteworthy that the discussions often represented a light bulb moment for many participants, who quickly questioned *why* they had not previously thought about sport as a context for child protection violations. During the course of discussion(s), most consultants identified formal and informal ways rights experts and the world of sport could collaborate moving forward.

### The journey to reporting – The 5 Rs

There were many critical points along the reporting journey identified by interviewees as either facilitating or hindering progress toward effective resolutions. The critical points are summarized as:

- **R**eadiness
- **R**ecognition
- Disclosure and **r**eporting
- **R**esponse
- **R**emedy.

#### Readiness

The first stage of the reporting journey is a sport organization’s readiness to actually engage with the reality of neglect, and/or sexual, physical, and psychological harassment and abuse against children in sport. If adults and authority figures in sport generally lack such preparedness in social contexts outside of and beyond sport, then this is likely to represent a significant barrier which may result in these persons not being ready to start the reporting journey in sport either. This stage of the reporting journey aligns with literature on trauma-informed approaches and practices in healthcare settings (Menschner and Maul, 2016), and may add a dimension to sport safeguarding frameworks that start with “recognize.” Readiness, which can also be thought of as cultural preparedness, precedes recognition. In the current study, this idea arose in the context of focus group participants experiencing a general *lack* of readiness with respect to child protection violations. Identified themes included:

- Fear- and Discomfort-related Avoidance/Aversion – “People do not want to think about violence against children, this is true from the level of the state right to the level of the person.” (Child Protection Expert)
- Reputational Risk – “Organizations are concerned that if they start talking about the issue, and start having policies and procedures, then they must have had a problem. This is a reputational risk and hence they do not go there.” (Human Rights Expert)
- Child and Athlete Development Considerations – A few respondents stated that based on their developmental stage, education/training, and life experiences, some children and adolescents are fundamentally incapable of interpreting abuse that happens to or around them. A number among athlete respondents recalled being young athletes brought into abusive sport relationships rapidly and without knowing what is expected or appropriate. If abuse is perpetrated by individuals who have a position of importance in relation to the child’s hopes and dreams in sport, e.g., a scout, a development coach, a person with access to higher level sport authorities and games, a child has a devil’s dilemma - risk everything in sport to stop the abuse or put up with the abuse to have a sport career. A number of athlete respondents said their love of sport withered with their abuse experiences.
- False Belief in the Absolute Purity of Sport – A few discussants from non-sport sectors shared that they struggled to believe that child protection violations occur in youth sport.

Many participants pointed to the need for education to address these challenges:

“If children and their caretakers are well-educated about grooming, emotional and physical abuse, and the impact it has on the brain and the victim’s future, they are far less likely to be victimized. If children and their caretakers are publicly, repeatedly reminded what to do if any abusive behaviours occur – including micro-aggressions – it makes it far more difficult for abusive individuals to muddy the waters, manipulate, play the victim when reports come in about their abuse, divide and conquer, offer favors to those who remain quiet or look the other way, offer opportunities to the athletes of those in power and so on.” (Researcher).

Lack of readiness individually and organizationally was called out as a contributor to repeated and at times unchecked child protection violations, increasingly serious violations as the athlete moves along their sports/child development pathway, and a developing sense of impunity by perpetrators and an overall delay in addressing the core issues.

#### Recognition

Sports’ and sport authority figures’ clear and shared understanding of what constitutes or may constitute a child protection violation is the nest step on the reporting journey. Study participants highlighted challenges related to recognition due to violations at times being normalized in sport contexts, particularly those that are non-sexual. One participant, an adult who had experienced abuse as a child in youth sport said plainly, “Athletes do not call their experience[s] abuse; it is just normal.”

The reasons for normalizing may be culturally specific, sport specific, and/or context specific. Mental or physical abuses against children may also be an accepted part of nuclear family or broader community economies. If players know and joke openly about various instances of abuse, there is no reason to expect a child being abused or any bystanders observing abuse (perhaps themselves victimized) would disclose informally to family, peers, or community affiliates (pastor, physician, etc.), or report formally through established grievance mechanisms. Here, abusive behaviors may be mistaken for a core part of sports culture.

Additional barriers which prevent people from recognizing child protection violations in sport, were identified as:

- Othering/Dissociating – “Abuse is seen as being problematic in ‘other’ cultures, religions, communities etc. not in your own and especially not in sport” (Safeguarding Expert)
- Image Management/Denial – “Nowadays it looks like sport tries to look as a microcosm with no problems which is just not true at all. By claiming that everything is ok the way it is right now just supports the idea that the problem might be bigger than we might even think.” (Athlete)

This extends to those working around the athlete: “Professionals must be informed and educated: how to detect, what to do when detected, what are the next steps?” (Practitioner).

Unique indicators of trauma in sport were discussed, including the fact that low energy or self-destructive behavior may appear differently in sport contexts. Physical pathologies and disease conditions (unintentional injury, dysregulated menstruation) and neurological impacts (poor attention and memory) were called out as signs of abuse that could show up in sports performance. Two questions related to these themes were asked aloud (but not answered) by numerous focus group participants: *How can those in sport be better supported to accurately see performance-related abuse indicators*? *If abuses are masked or altered by virtue of sports practice, what do we need to do differently in sport* versus *outside of it to improve recognition*?

#### Disclosure and reporting

There was a consensus among all focus group participants that the majority of child protection violations in their sport and/or in sport generally, are neither disclosed nor reported. “It is key to come back to the idea that the majority of abuse will not be reported so it’s key to build protective factors within the adult community and others with the understanding that reporting [systems have] many limitations” (Advocate).

The word “trust” was often used to describe young people’s unwillingness to come forward to either informally disclose or formally report abuse: absent a sense of trust in what or whom they might turn to, disclosures could not occur. A range of other considerations were identified, including:

- Terminology – “They need to know how to report, what words to use–the correct terminology, so that they can properly assert their rights to an abuse-free childhood.” (Sport Safeguarding Expert)
- Awareness – “In [an] elite athlete study, athletes asked ‘Were you assaulted? and Do you know where to report?’ Only 5% knew.” (Child Abuse Lawyer)
- Testing – “Children … often test the system first before they make a [formal] report.” (Practitioner)
- Mandatory Reporting – “Children are frightened to [formally] report as the law is too strict. This is to do with them not being able to talk through and share what was happening without it having to be reported straight away rather than them having time to talk to someone confidentially before having their situation reported.” (Practitioner)
- Intimidation and Fear – “[The term] ‘investigation’ equals formal equals scary.” (Athlete)
- Developmental Stage – “It was like trying to make adult decisions in a 10-year old’s body” (Sheldon, 2021, 95).

Many participants made the point that the onus should not be on the child to disclose or report. One athletic trainer said, “It is not right to ask or expect kids to report. This does not make sense. They can be taught to recognize, resist and report to increase their knowledge but not to increase their reporting.” (Trainer) Barriers related to the perceived consequences of reporting were also discussed, including:

- High Professional Costs – “When the penalties are high, e.g., derail or lose your career, get fired, others are reluctant to report.” (Athlete)
- High Personal Costs – “Athletes often say reporting often has wide-reaching negative impacts on the athlete and their families - including suicide.” (Child Abuse Lawyer)
- Fear of Retaliation – “From my experience as a former elite athlete, a retaliated-against whistleblower in my sport, a third party victim of sexual discrimination, and an activist who supports current athletes who report abuse in my sport, fear of retaliation is the number one problem that needs to be addressed. This is why you have Safe Sport investigations stalling and coaches who should be banned, not getting banned.” (Athlete)
- Obligation – “We need to prioritize the good welfare of athletes, so they do not feel they owe anybody to make a breakthrough in their careers.” (Practitioner)

Parental barriers to reporting were described as follows:

- Stigma – “One barrier for parents is the stigma of reporting, particularly if it concerns a boy child.” (Advocate)
- Cost/Benefit Analysis – “I think that parents think of the costs and benefits. We even had a parent … ask us to not report it for two weeks because they had a big championship coming up.” (Practitioner)
- Irrational Aversion – “Parents will move their kids to another program but not report.” (Practitioner)
- Unwitting Enabling – A handful of discussants described children being reluctant to tell parents especially when parents had invested a lot of time and money in getting their children to practice and to ‘special practices’ with perpetrators, and young athletes thinking their parents were receptive only to hearing about their athletic achievements.
- Parents as Victims of Grooming - There were several descriptions of how perpetrators groomed parents as well as players and/or players’ coaches and that parental grooming acted as a reporting barrier. One report stated, “the deeper the relationship with parents the greater the harm suffered by the child (Sheldon, 2021).”

#### Responding

Issues were identified regarding how athletes are treated by the process when reports are made. A key theme was onus of proof: “The victims feel unsupported by the various federations because they are deemed lying until proven otherwise” (Athlete). “A lot of times when athletes do talk, they are not believed and protected” (Lawyer), partly due to the passage of time which enables questions about recall accuracy.

Concerns regarding the ability of key individuals within sports organizations to respond effectively followed these themes:

- Second Chance – “Leaders and administrators and directors must take responsibility for each and every child participating in sport on their watch. They need to know that if they receive a report and give the coach or whomever a “second chance,” when the next report comes in and the next, they may well find themselves in conflict of interest because they are now in a position of negligence … A cursory glance at abuse cases in recent years will show them just how many leaders empowered with child safety have in fact protected the abusive individual and not the victims.” (Researcher)
- Capacity – “It is key to shore up capacity in this area; child protection services are commonly overwhelmed; in-sport concerns are challenging because most of the sports bodies do not have people with the skills and knowledge who know what to do.” (Practitioner)
- Willingness – Victims described feeling that they and/or their parents were actively discouraged from following up on a case with charges because of risk of retraumatizing others involved, perpetrator dismissal, or inadequate financial resources to proceed.

Participants highlighted non-sport experts’ limited understanding of sport as a barrier to effective response: “I have had firsthand experience that Statutory Bodies, including the Police and local government, have little or no understanding of the complexity of elite sport and therefore how to act upon safeguarding concerns” (Practitioner). There were also concerns regarding the relationship between sports organizations and other service organizations concerning case management: “There is a massive difference of opinion in terms of which concerns should stay within sport and those that should go out to be managed by other organizations” (Practitioner). This also extends to the role of the media: “There is a real issue of terrible professionalism [in the media]. For example, there was significant secondary damage caused by the media in one case because the media had tipped off an organisation that a report was going to be published. From that point on, the athletes were unsafe… some running for their lives” (Safeguarding Expert).

#### Remedy

The final stage of the reporting journey is the outcome. Healing must take place, justice must be done, and the organization must learn constructively from the case. Focus group participants often highlighted that effective remedy was not often achieved. For example, there were often negative outcomes for the person making the disclosure or report:

- Exclusion of Whistleblowers – “Inncreased protections for whistleblowers need to be in place so that those speaking out on abuse are not made to leave the sport afterwards - it is not their behaviour that is unacceptable.” (Advocate)
- Re-traumatization of Victims – “Of note, a lot of survivors have dropped out and stepped back due to the re-traumatization they experienced while setting up our advocacy organization. Rather than a growing army, it’s therefore more about targeted work *on behalf of* the survivor-army.” (Advocate)
- Need for Psychological Support – “Though this work is specific to sport, there is an underlying commonality in survivors and allies. There may be a huge cost for survivors to talk publicly, depending on where they are on their journeys. Some need therapy – and that is difficult if they are in the public arena.” (Advocate)

There were also concerns that appropriate outcomes had not been achieved regarding perpetrator punishment and subsequent sport access. In reference to the concept of perpetrator migration, one participant who had been abused as a youth athlete said, “In order to make change, there needs to be harsher penalties for those who verbally, mentally, physically and sexually abuse children. As of right now, a coach could sexually abuse an athlete, be banned from coaching in that sport and then start coaching in another sport. There needs to be full transparency as to why a coach is being suspended or banned.^2^”

Key concerns were lack of learning from previous cases, and disclosures not leading to any material change. Said a child abuse lawyer, “We need cases to be properly investigated such that organisations can critically learn the lessons. There is a clear gap between report and management that can only be closed by the learning loop.” An athlete reflected, “Having whistle blown on abuse I can honestly say I have not come across any individual or organization that is supporting or driving change around safeguarding in sport.”

Participants identified regularly and meaningfully asking sport participants about their well-being as a strategy for both abuse prevention and early intervention. Check-ins not only build mutual trust over a period of time but provide a sense of regular monitoring and empathic concern, while offering opportunities for athletes to express even low-level concerns. One advocate explained that if only someone would have asked the athlete more regularly how they were doing, they would have been able to see how happy or unhappy, validated or invalidated, safe or fearful they were in their sports environment and/or with their perpetrator.

Discussants identified many stages along the reporting journey at which a concern does not progress satisfactorily or where appropriate support is not provided.

### Influencing factors

Each stage of the reporting journey is influenced by a range of factors at the individual, relational, organizational and contextual level.

#### Individual factors

Interviewees pointed to a range of individual factors which may increase susceptibility to abuse and/or reporting behavior. “We need recognition that intersectionality, i.e., age, level, gender, disability, ethnicity including indigenous/tribal, homeless, LGBT, must be considered” (Safeguarding Expert). Some argued that sport should preferentially focus on those with additional vulnerabilities when undertaking child protection work. The central message was, start at the margins and work in. The argument here is that if an approach works with those at the margins who are rendered most powerless by common systems of oppression and privilege, then it is more likely to work other, less vulnerable groups. This ‘flips the script’ on the traditional top-down approach of designing strategies among mainstream subjects before subsequently adapting them for those at the margins.

Regarding the capacity to consent, “It is important to move beyond the concept of just ‘unwanted’ types of behaviors, because that word is somewhat irrelevant when some behaviors in these relationships may actually be wanted, but consent and the capacity to consent is however [not there]” (Athlete). Though typically applied to sexual relationships, consent is applicable to the full range of abuse in sport. As participants described, athletes may comply or acquiesce due to factors such as the normalization of the given behavior, the hierarchical culture of sport, and/or the ‘no pain, no gain’ mentality where harm is viewed as having instrumental effects on performance. These individual factors impact the reporting journey at every stage.

#### Relational factors

An athlete operates within a network of relationships with significant others, such as parents, friends, coaches, clinicians, fans, and teammates. At the heart of this relational influence is power. One athlete said, “It is all about power imbalances. It is a unique coach-athlete relationship. When this is combined with the extent of the elite athlete’s dream then these are all factors which make the sports environment uniquely vulnerable” (Athlete). The power of the elite performance dream also has a significant impact on parents, as in this exemplar quote: “I believe the abuse and grooming of the athlete and parents starts at an early age. In some sports parents receive a letter stating your child has potential at the age of 3 or 4. From then on the parents focus on the possibility of their child being great and become blind any other factors, including physical/emotional and sexual abuse” (Advocate).

Called out was a need to create a new normal that re-conceptualizes these relationships with athletes at the center. As for recalibrating relationships in sport, a safeguarding manager said, “We can address this by creating a new paradigm of relationships between all stakeholders in sports, re-building the chain of ‘dominance’ existing in sports – management, coaches and technical staff, athletes – by re-thinking the sports as an athlete-centered activity.”

#### Organizational factors

Participants highlighted the need for effective governance. This was both in terms of leadership and practice monitoring:

- Leadership – “We should have Child Safe Officers, complaints officers, member protection officers, but ultimately the CEO should be proactive and lead on these issues so everyone knows that the organization will do all they can to prevent issues occurring and provide a safe environment.” (Decision-maker)
- Senior Buy-In – “Power in international sport resides with the executive board and CEO. Without buy-in from sport leadership, change will not happen.” (Clinician)
- Open Door Policy – “Practices behind closed doors should not be happening. There should be a willingness at all times to have child caretakers, administrators, directors there as witnesses. Video cameras should be used not just to study practice, but to have a level of accountability at all times.” (Athlete)
- Sports Clubs – “Early access to children by perpetrators is often through the club or regional structures or local sport governing bodies. With low-level scrutiny, this is where coaches and trainers and scouts get hired. This is often where the first abuses begin and hence, where the first protections for children need to be located.” (Coach)

There were also a series of fundamental issues which speak to the culture of sport. These were viewed as creating a context in which disclosures would not be encouraged or managed effectively. “In the sport environment, autonomy is key. It is our own little realm, our domain, we rule over this independently, the primacy of protecting the integrity of the sport itself – protect the badge” (Athlete). In addition to the autonomy of sport, the culture of secrecy and silence was talked about: “The team dynamic that is taught to kids - which is a fake community but appears like a true one - namely the idea of what happens on the team stays on the team, needs to be openly questioned and halted” (Athlete).

Fundamental culture and systemic changes were seen as solutions: “We need culture change to view athletes as people first, and athletes second. We need to shift the focus from being purely on performance to well-being” (Advocate); “All sports clubs need to have better policies in place and easy access on how to report suspected abuse” (Athlete).

We identified promising examples from within sport which focus on situational change. One academic interviewed used a Situational Prevention Approach in sport and described promising early results and outcomes. Brackenridge’s work on activation states (Brackenridge et al., 2005) and research by Rhind and Owusu-Sekyere (2020) on developing a safeguarding culture were also called out.

Study participants gave examples of how adopting a child-centered approach has helped to change organizational culture. Terre des Hommes established children’s forums in 40 countries around the world. These forums are consulted on issues impacting children. The Breeze for Hope programme in Bolivia is another example discussed. Through identifying children as experts, they have achieved the highest national conviction rate for child sexual abuse in the world (95%). “Our child-directed governance structure makes our organization unique and disruptive. 30 teenage survivors of sexual violence sit on our board as fully voting members … As soon as we renovated our governance structure … the children experienced a gut-level sense of ‘Here, I matter and my voice matters’ … Straightaway our slogan became, ‘Nothing About us Without us.’ The youth we serve control our new performance dimension. Without them, we simply cannot perform at this level.” (Advocate).

#### Contextual factors

The final set of influencing factors were identified at the contextual level beyond the organization. This relates to governing bodies:

- National Governing Bodies – “The key influencers for Olympic sports are the Performance Directors. If they allow a coach or the management to behave in this manner because it brings medals that increase funding, the ends justify the means. Therefore, the Performance Directors need to be supported by the NGB in a change of policy that focuses on how these medals are won and does not decrease oversight of the sport when success arrives.” (Athlete)
- International Governing Bodies – “The only people who can make this work is the International Governing Body of each sport. They can insist on countries adopting their policies. Top coaches in every sport move around the world coaching in different countries and some move between disciplines and sports. An international course which has the best interests of the child/elite athlete at the heart of it would go some way to assist in this becoming a reality.” (Decision-maker)

It also extends beyond sport: “The government department responsible for the sport area can also be the one who drives the change, demands criteria which should be met to maximize the protection of athletes, and to create policies” (Athlete). Another important theme was the need for sports organizations to work in collaboration with other stakeholders, including “Public-private partnerships; partnership with civil society. Nothing is done alone. There is a need to link with others with shared values, synergistic capacities, common directions.” (Child Rights Expert). Across sports, these partnerships can help through knowledge sharing:

Cases of abuse need to be shared across jurisdictions so those involved in abuse are not able to simply move to other regions or jurisdictions without any mandatory background checks being available and performed. There should be mandatory reporting of findings to a central data base that it must be mandatory to cross check prior to any new hiring. (Safeguarding Manager).

There is also need for greater collaboration between organizations in sport and other related experts. Sport was described as a blind spot for child protection specialists write large: said one child rights expert, “There is general protection of children from violence – but for society – but no one is looking at sport.” A child protection expert followed up with: “The biggest challenge is that sport is not identified as a priority. It receives minimal attention within the broader child protection system.”

The need for an independent perspective was viewed as being important in terms of both reporting child protection violations and managing these concerns. “Athletes should be able to tell their story in a non-federation related organization. I think it would be good to have a professional (and full-time paid) ethics committee. Kind of ombudsman service for abuse. Together with local (club or province related) contact points” (Athlete). A safeguarding expert added, “There needs to be greater independence for issues to be dealt with. The National Governing body is often complicit in the abuse and therefore is conflicted in terms of its role of governance, particularly where it’s the only body with jurisdiction to sanction” (Safeguarding Expert).

The final contextual factor was the broader culture related to rights within the given context. “Every society differs because of the cultural differences, so the approach and understanding of every country is different” (Child Rights Expert); “We need to use local, context specific approaches through tailored responses across the globe” (Child Rights Expert).

#### e-Sports

Amid controversy related to ‘games’ versus ‘sports’ classification,’ a new context which was identified throughout this study as urgently needing our attention was eSports. Comprised of large audiences attending events in person and online, with many of the players and audience members being children and young poeple, eSports training and competition venues are vulnerable to child protection violations. Study participants discussed global eSport ‘athletes’ having entourages of coaches, physical trainers, physiotherapists, companions (for traveling to big stadiums) and managers; parents informally ensuring computer activities are appropriate to children; uneven player supervision where many players spend large proportions of the day in isolation improving their skills and rankings; player contracts to play in return for remuneration, share of prize monies, accommodation, gifts and so on; but no clear governing body or international federation overseeing protections for everyone involved.

In sum, eSports is a rapidly growing, monied environment in which children and youth play for high stakes. The potential for abuse is high: there is little to keep predators from having access to players, little preventative work with players about human rights and safeguards, a high risk of child exploitation sexually, physically, emotionally and financially; an industry that is growing faster than it can “get its safeguarding house in order.” This presents an opportunity to engage with the leaders of the eSports organizations to work from the ground up on international safeguards and child protection.

## Discussion

Overall, the disclosure of child protection violations is a complex and dynamic process where individual, relational, organizational and contextual factors interact to influence progress toward effective outcomes, including healing. The current study highlights themes that reinforce and add to prior work in this field, from new stakeholders’ perspectives.

First, we acknowledge as others have, that one size does not fit all when it comes to child protection in diverse global sport settings; international comparisons are important, and individuating child protection approaches by context is also key (Lang and Hartill, 2014). Even as we review foundational rights-related documents, it is important to heed scholars’ calls for their decolonization. While “in a child’s best interests” is becoming a universal concept, its roots are still embedded in Western understandings and perspectives (Holzscheiter et al., 2019; Faulkner and Nyamutata, 2020). In the world of sport, we are acclimatizing ourselves to the use of children’s rights to frame our safeguarding work (Mountjoy et al., 2015). We certainly must be more aware of the ongoing critiques of human rights developments and how we may embed them into our work, as we global contexts.

Second, the importance of bringing athletes’ and survivors’ voices to the forefront of safeguarding work cannot be overstated (Guiora, 2020; Tuakli-Wosornu et al., 2022b; Bekker and Posbergh, 2022; Lang, 2022). This is especially important when athletes identify as having an intellectual, physical, or other type of impairment, as being part of the LGBTQ+ community, being racialized/minoritized, and/or being a child or youth (Kirby et al., 2008; Mountjoy et al., 2016; Tuakli-Wosornu et al., 2020; Hartill et al., 2021; Rhind et al., 2021; Bekker and Posbergh, 2022). A key concept that emerged from the study was the importance, power and efficiency of designing from the margins in, i.e., starting in sport settings where risk compounds. Interestingly, there was no element of critical thinking that emerged, e.g., directly challenging the autonomy of sport organizations, identifying cultural barriers on standard reporting pathways, or even thinking that the reporting framework might be best used in Western cultures but not others. One solid outcome therefore, is the observation that global sport must proactively find ways to ensure all children of all cultural and sport contexts are safe.

The 5 R framework – Readiness, Recognition, disclosure and Reporting, Response and Remedy, outline critical points on the reporting journey. Readiness is a new concept that emerged out of the realization that we were talking with child protection experts who had little to no readiness to engage with child protection violations in sport. Individual athletes may not understand, nor want to understand, what interpersonal and/or collective violence is. This can be addressed through meaningful education at many levels of sport. Organizationally, our use of a primer (Figure 1) helped the child protection people to see sport in a new light – and sport too needed their child protection expertise. This clearly showed that unless there is readiness to engage, rights will not be protected, and violations will continue. Absent readiness, athletes may experience increasing harm over their lifetimes (Sheldon, 2021).

For sport, recognition includes taking a definitive step away from the often-entrenched habit of assuming gain from pain and ‘explaining away’ otherwise unacceptable displays of interpersonal and collective violence as part a ‘normal’ part of sport culture (David, 1998, 2004; Hartill et al., 2021). Sport needs to become – or return to, a more inclusive, and overall more respectful and dignified space (David, 2004; Tuakli-Wosornu et al. 2021a,b; Tuakli-Wosornu and Kirby, 2022). Recognition is a major tool in the toolbox for child protection and safeguarding in youth sport.

Perhaps for the first time ever, we see the reasons why disclosure and reporting are together the weakest link in the safeguarding chain. Reasons for this included lack of trust/confidence in people and systems, children’s propensity to informally test the system before being comfortable formally using it, clear preference for informal disclosure over formal reporting and ‘investigation,’ and lack of clarity of where to disclose or report; these reinforce data presented elsewhere (Hartill et al., 2021; Rhind et al., 2021). Barriers to informal disclosure and formal reporting were clearly much more nuanced than one might have assumed. This is helpful to those in policy and practice who seek to strengthen this one part of their safeguarding framework. It will be possible to do strategic work around, for example, barriers to reporting for parents, coaches, and other adults (Verhelle et al., 2022).

If an issue is raised, it must be responded to. If not, the whole safeguarding framework cannot work. Those raising the issues must trust that they will be heard and that there will be some outcome. The mantra of “listen, record, report” still works. Even if the child protection people do not understand sport, they can be major players in bringing their skills to sport safeguarding. In other words, the safeguarding people in sport do not have to go it alone but can work with knowledgeable others outside of sport to build overall child protection capacity. Again, having strong leaders committed to protecting children’s rights will help to ensure that organizations stay true to the safeguarding goals. What also came clear is that supports are needed for those in the reporting pathway, particularly the victims. That support may need to take many forms (e.g., psychological, financial, legal) and may be needed beyond any case management closure.

From the data, it seems that remedy is the least understood in sport. Often, there are difficulties, sometimes long-term, for the whistleblowers, the victims, and the organizations. Often there are few for the perpetrator, if any. Safeguarding work in sport has not shone the light too brightly on remedy but it is a critical but much understood element of the safeguarding framework. Identifying remedy as an area of great concern should shift some research attention toward it.

Overall, these data add significantly to a growing cannon of sport safeguarding literature by contributing a qualitative analysis specifically examining the abuse reporting pathway for children who participate in sport. The constructivist paradigm used, which adopts a transactional and subjectivist epistemology (Guba and Lincoln, 2005), enabled a more textured understanding of stakeholder priorities within the context of their dynamic lives and experiences. Consistent with prior work (David, 1998; David, 2004; Pinheiro, 2006; Vertommen et al., 2016; Rhind and Owusu-Sekyere, 2020; Sheldon, 2021), our data indicate that when it comes to safeguarding, the rights of a child and the rights of an athlete appear to be far apart. What is in the child’s best interest in sport would have safeguarding included, if not central to the ethics and integrity issues such as doping, match fixing, and cheating. However, when we asked those responsible for implementing a broad continuum of rights and responsibilities in relation to the care of a child, we found little if any indication that they knew about sport or that sport was contributing the rights-based discussions. Consistent with trauma-informed work outside of sport, one additional dimension our qualitative data added was the critical importance of “readiness,” when establishing safe sport environments–it is insufficient to start at “recognize” (Menschner and Maul, 2016; Tuakli-Wosornu et al. 2022a,b).

Of note, for pragmatic reasons, we mostly focused on sexual abuse in this manuscript. Though it is not and we should not be using this concept as shorthand for any other form of abuse, we do not yet have an agreed upon coherent set of definitions that we use across sport. Our use of the term interpersonal violence works for researchers and even policy makers but does not resonate with those doing child protection work inside or outside sport. Sport will have to come to terms with this in short order. This lack of specificity could lead, inadvertently, to those in sport thinking that they are fully protecting children if they protect them from sexual abuse alone. Nothing could be further from the truth. This has not helped us much in protecting *all* children’s rights. We must aim further –child protection is not ‘achieved’ until all children’s rights are protected.

### Study limitations

Limitations of this work include restricting analysis to children, rather than including both children and adults. Additionally, though qualitative approaches to understanding and/or characterizing abuse reporting behaviors and frequencies, the number of stakeholders becomes limited. A mixed methods study, where a quantitative survey was disseminated and resulted to complement qualitative data, may have opened the door to increased number of respondents. As above, we mostly focused on sexual abuse in this manuscript. Finally, as Franklin and Nyamutata aptly argue, this and other analyses that stand on current rights-based documents and policies cultivated within a Western-centric perspective, may inadvertently exclude those for whom a Westernized conception of human rights is misaligned with local ways of knowing, being, and interacting. Our foundation for this analysis is the UNCRC – Section 19. Though well-articulated in the UN conventions, children’s rights are variously interpreted across time and space and “practices of agency” and cultures, including the culture of sport. The UNCRC ‘imposed a universal notion of what it is to be a child; it has prescribed and embedded what the substance and scope of children’s rights should be.” When considering who is a child and what is “in their best interests,” the UNCRC takes a distinctly Western view. Those with different cultural views may find themselves limited by resources, language, tools and opportunities in participating in the critical efforts to decolonize the field of children’s rights and their protections. Though beyond this manuscript’s scope, the colonial legacy of these treaties and what that means for sport was not addressed and limits the work’s universal applicability.

Due to its accessibility and pragmatism, the *International Safeguards for Protecting Children in Sport* was selected as our starting point and conceptual frame. We acknowledge, however, that these safeguards capture only the four most recognized forms of abuse, and tend to focus on sexual abuse alone. As we seek understanding of existing child protection guidelines for sports-involved youth, it is incumbent on us to have a critical eye with regard to the original contexts in which they were created and note associated limitations. Protecting children from only the four most recognized forms of violence does not ensure they are protected from all forms, or even the most harmful or prevalent forms.

## Recommendations for future practice and research

From the foregoing, we first recommend that the Reporting Journey and Influencing Factors (the 5Rs) be used to link and prioritize projects on children, sport and safeguarding. Table 1 shows how the disclosure journey and influencing factors interact to highlight a range of potential questions that can guide future work by generating insights on the growing consensus to embed human rights in sport.

**Table 1:** Reporting journey and influencing factors.

Reporting journey	Influencing factors			
	Individual	Relational	Organizational	Contextual
Readiness	How do inidividual talk about rights in general and what implications does this have for sport?	How do stakeholders talk general and what implications does this have for sport?	What are the inidicators that an organizaton is ready make progress on respecting rights?	What are the inidicators that a community respects rights?
Recognition	How do we raise awarness?	How do we raise awarness	How do we encourage buy-in	How do we engage non-sport stakeholders
Reporting	How can we support athletes to report?	How can we support bystanders to report?	Which organizational factorrs facilitate reports?	How can sport collaborate with other agencies to promote reporting?
Response	How can we support athletes during investigations?	How can we support significant others during investigations?	What facilities effective case management?	What is the feasibility of independent case management in sport?
Remedy	How do we support athletes with lived experience to engage in this work?	How do case outcomes impact others in sport?	How can organizations learn from case?	How can sport learn from other sectors?

A second recommendation is that a global safety net environment be established to build a response and remedy system that will have common core components globally but with applications varying from region to region around the world. This would be a safety net culture of social norms and behaviors, broadly informed by the *International Safeguards for Protecting Children in Sport*, core components of integrity issues in sport, adverse child experiences (ACEs) framework and other biological/physiological factors found in public health and other trauma-focused fields, athlete-centered justice, and our new understanding of the reporting journey and influencing factors.

A third recommendation is working with specific child-focused partner agencies and organizations to enhance child protection and safeguarding work in sport so safeguarding is conceptualized as one part of a holistic view of children’s rights. This could lead to sport organizations working with the Convention on the Rights of the Child Committee (CRC) on a review of current mandates on recreation and play (to move this toward sport), and enhanced mandates for child protection violations disclosures.

The final recommendation is that safeguarding be brought to unregulated (sport) environments, e.g., eSports, children on elite performance pathways (music, dance, sport and other performance industries), child participation away from scrutiny, in organizations that do not get funding, ‘one-man bands’ (e.g., martial arts, sport scouts), unregistered teams and some fitness industry activities. These environments were touched on briefly during focus group discussions but represent an under-served area with weak or absent safeguarding structures.

## Conclusion

It is important that interpersonal violence in sport be unanimously conceived of as human rights violations (on par with education- and healthcare-related violations) so the status of, urgency surrounding, and resourcing related to athlete safeguarding can be escalated in influential circles. As this happens, sport may become a safer and more enjoyable environment for all participants, including sports-involved children and youth.

## Author contributions

YT-W, SK, AT, and DR conceptualized and designed this manuscript. YT-W and DR drafted the initial manuscript. All authors contributed to the article and approved the submitted version.

## Funding

The authors thank Oak Foundation for supporting this project.

## Conflict of interest

The authors declare that the research was conducted in the absence of any commercial or financial relationships that could be construed as a potential conflict of interest.

## Publisher’s note

All claims expressed in this article are solely those of the authors and do not necessarily represent those of their affiliated organizations, or those of the publisher, the editors and the reviewers. Any product that may be evaluated in this article, or claim that may be made by its manufacturer, is not guaranteed or endorsed by the publisher.

## Footnotes

^1^Here, we use the World Report on Violence and Health’s definition of violence: ‘The intentional use of physical force or power, threatened or actual, against oneself, another person, or against a group or community, that either results in or has a high likelihood of resulting in injury, death, psychological harm, maldevelopment, or deprivation.’ Sport can also be home to self-directed violence (i.e., violence against oneself), but in the present report, we see self-directed violence as a consequence of interpersonal violence and so focus on interpersonal. For more context, the World Report on Violence and Health focused on topics including child abuse and neglect, youth violence and sexual violence. Health and social impacts were discussed along with risks factors, protective factors, and prevention initiatives. There was a great expansion of research on violence and health afterwards, one of which, is a clearing house of information that might be a useful network for researchers on violence and sport: *Violence Info*. This is an update of the World Health Organization Violence Prevention Information System, an interactive knowledge platform of scientific findings on violence (Krug et al., 2002).

^2^Of note, this is not the case in all countries.
